# Inequalities in the demand for dental services in Brazil: associated factors in the 2023 National Oral Health Survey

**DOI:** 10.1590/1980-549720260027.supl.1

**Published:** 2026-07-20

**Authors:** Fernanda Alencar Rodrigues Pereira, Ana Paula Alves Távora de Oliveira, Maraysa Costa Vieira Cardoso, Christina Cesar Praça Brasil, Geraldo Bezerra da Silva, Mirna Albuquerque Frota, Ana Livia Torres de Sousa, Antonio Augusto Ferreira Carioca

**Affiliations:** I Universidade de Fortaleza, Post-Graduate Program in Collective Health – Fortaleza (CE), Brazil.

**Keywords:** Health inequalities, Dental care, Dental health surveys, Dental health services

## Abstract

**Objective:**

To analyze sociodemographic factors associated with the use of dental services in Brazil, based on data from the National Oral Health Survey – SB Brasil 2023.

**Methods:**

This cross-sectional, population-based study used data from 40,489 individuals aged 18 years or older. The dependent variable was the use of dental services in the previous year. Analyses accounted for the complex sampling design, including weighting and design corrections. Descriptive and bivariate analyses (χ^2^ test) were conducted, followed by multiple logistic regression to estimate odds ratio (OR) and 95% confidence intervals (95%CI).

**Results:**

Approximately 58.8% of respondents reported having used dental services in the previous year. A higher likelihood of use was associated with being female (OR=1.44; 95%CI 1.20–1.74), having higher educational attainment (complete higher education: OR=3.16; 95%CI 2.01–5.00), and higher income (>10 minimum wages: OR=2.79; 95%CI 1.63–4.78). Conversely, increasing age (OR=0.99; 95%CI 0.98–0.99) and residence in state capitals (OR=0.70; 95%CI 0.59–0.83) were associated with a lower likelihood of using dental services.

**Conclusion:**

Inequalities in the use of dental services persist in Brazil and are influenced by educational attainment, income, and vulnerability-related factors. These findings highlight the need for public policies aimed at reducing structural barriers and expanding equitable access to oral health care.

## INTRODUCTION

Oral health is understood as the maintenance of the functional and structural state of the mouth, teeth, and orofacial structures. It encompasses not only the performance of essential activities, such as speaking, eating, and breathing, but also relates to psychosocial dimensions, including self-confidence, well-being, and the capacity for social interaction, thereby directly impacting individuals’ quality of life throughout the course of their lives^1,2^.

Since 2005, Brazil has made strides in organizing oral health care through the implementation of the National Oral Health Policy (PNSB)^3^, known as "Brasil Sorridente" (Smiling Brazil). Instituted in 2004, this policy aims to promote, prevent, and restore the population's oral health by reorganizing, reorienting, and enhancing the quality of the services offered, thereby expanding access to dental treatment through the Unified Health System (SUS) for people of all age groups. Above all, this policy seeks to combat inequalities in care by broadening the population's access to comprehensive dental services, with a particular emphasis on primary health care (PHC)^4,5^.

It is worth noting that health surveillance constitutes one of the guiding principles of the PNSB^3^; specifically, the utilization of epidemiological data to guide the planning of oral health initiatives stands as one of the policy's fundamental pillars. Situated within this context is the National Oral Health Survey SB Brasil 2023^3^, the data analysis of which enables the understanding and identification not only of the prevalence profile of oral diseases but also of the sociodemographic characteristics of both users and non-users of these services, thereby pinpointing the factors associated with the seeking of dental care^4,6^. By fostering improvements in access to oral health care, the PNSB marks a departure from a care delivery model characterized predominantly by curative and mutilating practices. Brazilian scientific literature^7–11^ and national surveys such as the PNSB and the National Health Survey (PNS) broadly and consistently document inequalities in access to dental services in the country, highlighting a persistent pattern of higher use among individuals with higher income and educational attainment, women, and users of the private sector. This reality stands in stark contrast to the lower levels of access to these services observed among low-income older individuals and those in situations of greater social vulnerability, even despite the existence of a universal health system^3,12^.

The 2019 PNS^12^, particularly revealed significant socioeconomic and regional inequities in the use of dental services, reinforcing the role of social determinants in the organization of oral health care in the country. It is worth noting, however, that much of the available information relies on data predating the COVID-19 pandemic or on general health surveys rather than those specific to oral health; this limits our understanding of inequalities and the demand for dental care within a context of profound social and organizational changes in service delivery.

It is evident that inequalities related to the socioeconomic factors sex, age, and regional structural barriers influence the demand for dental services in the country^3,9^. Nevertheless, the impact of direct markers of social vulnerability, such as the receipt of social assistance benefits as well as less obvious territorial pattern such as the disparities between state capitals and municipalities in the interior, remain largely unexplored.

In this regard, by utilizing data from SB Brasil 2023^3^ and analyzing the demand for dental services during the 12 months preceding the interview, the present study updated the diagnosis of oral health inequalities in Brazil and expanded the analysis of sociodemographic factors associated with the demand for care. By incorporating contemporary markers of social vulnerability, such as the receipt of the Continuous Cash Benefit (BPC)^13^; the study moves beyond classic analyses on the basis of solely income and education, engaging with evidence derived from analyses of previous editions of the SB Brasil survey and the 2019 PNS. Furthermore, the results indicate a lower seeking of services in state capitals, a finding that challenges traditional interpretations regarding service supply and access and that suggests the persistence of structural barriers even in contexts characterized by a higher concentration of services. Thus, this study contributes to refining the debate on equity and provides empirical evidence to inform the improvement of public policies within the scope of SUS.

However, there remains a gap in recent studies that update these findings based on the new national oral health survey, particularly those offering broader analytical scope and adjusting for emerging social variables, such as income transfer programs, the interplay of social and economic determinants, and disparities between state capitals and municipalities in the interior^14–17^. Against this backdrop, the present study aimed to analyze the sociodemographic factors associated with the demand for dental services in Brazil, according to data from the SB Brasil 2023 survey.

## METHODS

### Study design

This was a cross-sectional, population-based epidemiological study using a quantitative approach, developed on the basis of an analysis of microdata from the National Oral Health Survey SB Brasil 2023.

### Context (Setting)

SB Brasil 2023 was conducted through a partnership between the Ministry of Health and the Department of Social and Preventive Dentistry at the Federal University of Minas Gerais (UFMG), with technical-scientific execution carried out between 2017 and 2024. It consisted of a household-based study, with data collection taking place from March 2022 to March 2024. Field teams were composed of dentists (epidemiological examinations), oral health technicians or assistants (data recording), and community health agents (household enumeration).

The survey adhered to recommendations from the World Health Organization (WHO), utilizing stratification by index age groups (5, 12, 15–19, 35–44, and 65–74 years). Fifty-three geographic domains (state capitals, states, and the Federal District) were considered, from which census tracts and households were randomly selected. The final sample comprised approximately 50,800 participants, who underwent both interviews and oral examinations.

Interviews were conducted in person within the selected households by trained and standardized teams using a structured questionnaire. This instrument included questions regarding the demographic and socioeconomic profiles of the households, such as ownership of durable goods, family income, internet access, and receipt of social program benefits, as well as individual participant characteristics, including age, sex, color or race, and educational attainment. The instrument also investigated access to and utilization of oral health services, the occurrence of self-reported oral morbidity (toothache and facial pain), and self-perception of oral health and its impact on daily life. Prior to the commencement of fieldwork, all professionals participated in training sessions conducted by the research coordination team. These workshops covered participant identification protocols, the standardization of codes and criteria for oral examinations, and the use of the electronic data collection system. Furthermore, periodic monitoring was conducted to ensure the quality of data collection and the consistency of the data obtained.

It should be noted that further methodological details are available in the previously published electronic material titled "SB Brasil 2023: National Oral Health Survey: final report"^6^.

### Participants

For the present study, only individuals aged 18 years or older were considered, totaling 40,489 participants with valid data for the dependent variable "seeking dental care in the past year". The analysis was conducted while preserving the complex sampling design of the original survey, incorporating sample weights, strata, and clusters.

### Variables

The dependent variable was the search for dental services, which consisted of the question: "In the last year, did you seek out a dental office, oral health service, or dentist/oral health team to receive care"? The response options were: "I didn't seek care"; "I sought care but wasn't seen"; "I sought care but was scheduled for another day/another location"; "I sought care and was seen"; and "Don't know/Didn't answer". For analytical purposes, the variable was operationalized dichotomously; responses of "I sought care but wasn't seen", "I sought care but was scheduled for another day/another location", and "I sought care and was seen" were classified as "Yes," while the response "I didn't seek care" was classified as "No". Responses of "Don't know/Didn't answer" were treated as missing data.

The independent variables included: geographic region (North, Northeast, Southeast, South, Central-West); place of residence (state capital/interior); sex (male, female); age (in years); self-reported color/race (White, Black, Brown, Asian, Indigenous); educational attainment (no schooling/incomplete elementary education, complete elementary education, high school, and higher education); and family income in minimum wages (MW) (Up to 1 MW, >1 to 3 MW, >3 to 10 MW, and >10 MW). Variables regarding participation in government social programs were also included and considered separately: receipt of the Bolsa Família Program benefit (No, Yes); receipt of the Continuous Cash Benefit (No, Yes); and participation in other income transfer programs (No, Yes). Responses of "Don't know/Didn't answer" were treated as missing data.

### Statistical methods

Analyses were performed using Stata^®^ 17.0, incorporating sample weights and corrections for the complex survey design. Descriptive analysis was conducted to characterize the sample, followed by bivariate analysis (Pearson's χ^2^ test). Subsequently, multiple logistic regression was applied using a hierarchical entry of variables, based on the conceptual model proposed by Victora et al.^18^, to estimate crude and adjusted odds ratios (OR) with 95% confidence intervals. A hierarchical conceptual model was adopted to analyze factors associated with the demand for dental care. At the most distal level (Model 1), individual demographic characteristics (age and sex) were included; at the intermediate level (Model 2), socioeconomic variables (educational attainment, BPC receipt, and family income) were considered; and at the most proximal level (Model 3), contextual variables related to place of residence (major regions of the country and residence in a state capital) were included. Variables were entered into the model sequentially; those demonstrating statistical significance were retained in the subsequent levels, in accordance with the hierarchical model.

### Ethical aspects

The study adhered to ethical principles for research involving human subjects, having been approved by the National Research Ethics Commission (Conep) on July 3, 2021 (CAAE 34497120.6.3001.0008; Opinion No. 4.823.054).

#### Data Availability Statement:

The entire dataset supporting the results of this study is available upon request to the Ministry of Health. To access the SB Brasil databases, it is necessary to agree to the terms of commitment and provide the information requested by the Ministry of Health.

## RESULTS

Of the participants analyzed, 58.8% reported having sought dental services within the last 12 months. Table 1 presents the distribution of this use according to various sociodemographic variables, highlighting patterns of inequality in dental care in Brazil. Utilization was associated with city of residence, age, sex, educational attainment, family income, and receipt of the Continuous Cash Benefit (BPC).

**Table 1:** Distribution of seeking dental services according to sociodemographic characteristics. National Oral Health Survey (2023). Brasil, 2026.

Variables		Did not seek services (41.2%)	Sought services (58.8%)	p-value	Total
Major regions					
	North	8.1 (6.6–9.9)	7.6 (5.8–10.0)	0.091	7.8 (6.4–9.5)
	Northeast	27.0 (23.5–30.8)	25.9 (22.1–30.2)		26.4 (23.1–29.9)
	Southeast	42.1 (36.6–47.8)	42.8 (36.3–49.7)		42.5 (37.0–48.3)
	South	16.5 (13.7–19.9)	14.1 (11.5–17.3)		15.1 (12.7–17.9)
	Central-West	6.3 (4.9–7.9)	9.4 (6.5–13.5)		8.1 (6.0–11.0)
	City of residence, capital	26.0 (22.8–29.4)	22.1 (19.1–25.4)	0.014	23.7 (20.9–26.7)
	Age, years	48.1 (47.1–49.2)	43.5 (42.6–44.3)	<0.001	
Sex					
	Male	41.9 (38.7–45.1)	33.2 (30.9–35.7)	<0.001	36.8 (34.9–38.7)
	Female	58.1 (54.9–61.3)	66.8 (64.3–69.1)		63.2 (61.3–65.1)
Color or race					
	White	42.2 (38.3–46.2)	44.0 (40.1–47.9)	0.446	43.2 (39.8–46.7)
	Black	14.5 (12.2–17.1)	12.3 (10.6–14.2)		13.2 (11.6–15.0)
	Asian	1.1 (0.7–1.6)	1.2 (0.8–2.0)		1.2 (0.8–1.7)
	Brown	40.4 (36.6–44.4)	41.0 (37.4–44.7)		40.8 (37.5–44.1)
	Indigenous	0.4 (0.2–0.8)	0.3 (0.2–0.6)		0.3 (0.2–0.6)
Continuous Cash Benefit					
	No	83.1 (79.4–86.3)	88.2 (84.7–91.0)	0.013	86.1 (83.1–88.7)
	Yes	9.4 (7.3–12.0)	6.0 (4.7–7.6)		7.4 (6.1–8.9)
Bolsa Família Program Benefit					
	No	75.5 (72.1–78.7)	76.7 (72.6–80.4)	0.667	76.2 (73.0–79.1)
	Yes	17.7 (15.4–20.3)	17.6 (14.9–20.6)		17.6 (15.6–19.9)
Welfare benefit from other government social programs					
	No	79.8 (76.2–83.0)	81.2 (77.7–84.3)	0.648	80.6 (77.7–83.2)
	Yes	13.2 (11.1–15.7)	12.9 (10.7–15.4)		13.0 (11.2–15.0)
Highest level of education					
	No schooling/incomplete elementary education	34.5 (31.4–37.8)	20.1 (17.8–22.4)	<0.001	26.0 (23.7–28.4)
	Complete elementary education	10.1 (8.4–12.0)	8.3 (6.7–10.2)		9.0 (7.9–10.3)
	High school	42.0 (38.6–45.4)	47.8 (44.1–51.5)		45.4 (42.6–48.3)
	Higher education	13.4 (10.5–17.0)	23.9 (21.0–26.9)		19.6 (17.0–22.5)
Family income					
	Up to 1 MW	27.1 (23.1–31.6)	24.3 (20.3–28.9)	0.002	25.6 (21.9–29.5)
	>1 to 3 MW	52.3 (48.2–56.4)	46.7 (41.7–51.8)		49.0 (45.3–52.7)
	>3 to 10 MW	20.0 (16.0–24.7)	26.9 (23.1–31.2)		24.1 (21.0–27.6)
	>10 MW	0.6 (0.4–0.9)	2.0 (1.3–3.2)		1.4 (0.9–2.1)

95%CI: 95% confidence interval. Pearson's χ^2^ test for categorical variables. Data weighted and adjusted for complex sample design.

Source: Brasil, SB Brasil 2023.

In Table 2, residents of the Central-West region demonstrated a higher likelihood of seeking care (OR=1.59; 95%CI 1.04–2.44) compared to those in the North region; however, residing in state capitals reduced this likelihood (OR=0.80; 95%CI 0.68–0.96). Age (in years) decreased the odds of the outcome (OR=0.98; 95%CI 0.98–0.99), while female sex increased the likelihood of seeking care (OR=1.44; 95%CI 1.21–1.73) relative to men. The variable of color or race was not associated with the outcome.

**Table 2:** Bivariate analysis of factors associated with seeking dental care: National Oral Health Survey 2023. Brasil, 2026.

Independent variables		Dependent variable: seeking dental care		
		OR	95%CI	
Major regions				
	North	Reference		
	Northeast	1.01	0.72	1.44
	Southeast	1.07	0.74	1.57
	South	0.90	0.74	1.57
	Central-West	1.59	1.04	2.44
City of residence, capital		0.80	0.68	0.96
Age (years)		0.98	0.98	0.99
Sex				
	Male	Reference		
	Female	1.44	1.21	1.73
Color or race				
	White	Reference		
	Black	0.82	0.65	1.02
	Asian	1.12	0.61	2.07
	Brown	0.97	0.82	1.16
	Indigenous	0.74	0.33	1.70
Continuous Cash Benefit				
	No	Reference		
	Yes	0.60	0.42	0.86
Bolsa Família Program Benefit				
	No	Reference		
	Yes	0.97	0.78	1.23
Welfare benefit from other government social programs				
	No	Reference		
	Yes	0.95	0.74	1.22
Highest level of education				
	No schooling/incomplete elementary education	Reference		
	Complete elementary education	1.42	1.06	1.90
	High school	1.96	1.61	2.39
	Higher education	3.08	2.37	3.99
Family income				
	Up to 1 MW	Reference		
	>1 to 3 MW	1.00	0.78	1.27
	>3 to 10 MW	1.50	1.12	2.01
	> 10 MW	3.96	2.34	6.71

Crude odds ratios (OR) with respective 95% confidence intervals (95%CI), obtained via bivariate logistic regression. Analyses were weighted and adjusted for the complex sampling design of the National Oral Health Survey – SB Brasil 2023.

Source: Brasil, SB Brasil 2023.

Notably, receipt of the BPC significantly reduced the likelihood of seeking care (OR=0.60; 95%CI 0.42–0.86), whereas the Bolsa Família program and other benefits showed no statistical association. Furthermore, educational attainment revealed a significant gradient: completion of high school (OR=1.96; 95%CI 1.61–2.39) and completion of higher education (OR=3.08; 95%CI 2.37–3.99) significantly increased the likelihood of seeking care compared to those with no formal education. Family income followed a pattern similar to that of educational attainment: individuals with a family income of more than 10 minimum wages were nearly four times more likely to seek care (OR=3.96; 95%CI 2.34–6.71) compared to those with a family income of up to 1 minimum wage.

Table 3 shows that in the final model, factors independently associated with a higher likelihood of seeking dental services were: female sex (OR=1.54; 95%CI 1.21–1.96); higher educational attainment, particularly higher education (OR=1.90; 95%CI 1.37–2.63); and family income exceeding 10 minimum wages (OR=3.49; 95%CI 1.97–6.17). Conversely, age in years (OR=0.99; 95%CI 0.98–0.99) and residing in capital cities (OR=0.69; 95%CI 0.57–0.84) were associated with a lower likelihood of seeking dental services.

**Table 3:** Final logistic regression model of factors associated with seeking dental care. National Oral Health Survey 2023. Brasil, 2026.

		Dependent variable: seeking dental care								
		Model 1 (sex and age)			Model 2 (model 1 + education level, income and BPC)			Model 3 (model 2 + major regions + city of residence)		
Independent variables		OR	95%CI		OR	95%CI		OR	95%CI	
Age (years)		0.98	0.98	0.99	0.97	0.98	0.99	0.99	0.98	0.99
Sex										
	Male	Reference			Reference			Reference		
	Female	1.45	1.20	1.75	1.54	1.20	1.96	1.54	1.21	1.96
Highest level of education										
	No schooling/ incomplete elementary education				Reference			Reference		
	Complete elementary education				1.18	0.82	1.71	1.20	0.84	1.73
	High school				1.38	1.08	1.76	1.42	1.11	1.82
	Higher education				1.84	1.34	2.55	1.90	1.37	2.63
Family income										
	Up to 1 MW				Reference			Reference		
	>1 to 3 MW				0.97	0.77	1.23	1.02	0.82	1.26
	>3 to 10 MW				1.34	0.98	1.84	1.47	1.08	1.98
	>10 MW				3.03	1.70	5.41	3.49	1.97	6.17
Continuous Cash Benefit										
	No				Reference			Reference		
	Yes				0.71	0.45	1.14	0.71	0.44	1.14
Major regions										
	North							Reference		
	Northeast							1.07	0.70	1.65
	Southeast							0.96	0.61	1.50
	South							0.76	0.47	1.22
	Central-West							1.40	0.84	2.34
City of residence, capital								0.69	0.57	0.84

Adjusted odds ratio (OR) and 95% confidence intervals (95%CI), based on a hierarchical model. The variables were mutually adjusted according to social determination blocks. Analyses were weighted to account for the complex sampling design of SB Brasil 2023.

Source: Brasil, SB Brasil 2023.

## DISCUSSION

The findings of this study underscore the persistence of structural inequalities in the demand for dental services in Brazil, even in the face of institutional advancements promoted by the National Oral Health Policy. These results align with reports from various authors who highlight the role of social determinants of health in the patterns of dental service use, particularly in countries with universal yet unequal health systems, such as Brazil^15,17,19^.

The figure of 58.8% of respondents who reported seeking care within the past 12 months should be interpreted as an indicator of service-seeking behavior, conditioned by perceived needs and structural barriers rather than as a metric of health system coverage, given that no normative targets have been established for this specific outcome in Brazil^6^. This percentage should be understood as a descriptive indicator of care-seeking behavior, shaped by perceived needs, structural barriers, and individual and contextual characteristics. Similar results were observed in the 2019 PNS and in analyses of the SB Brasil 2010 survey^12,20^, which demonstrated that a significant portion of the population does not regularly seek dental care, even within a universal health system^7,9^. The unequal distribution across regions, despite not reaching statistical significance, reveals substantial concentrations of demand in the Southeast and Northeast, reflecting both population density and the historical structure of service allocation in the country. Recent studies reinforce that the geographic distribution of services and installed capacity vary significantly across regions, directly impacting access to and utilization of oral health care^7,15^.

A noteworthy and counterintuitive finding was the lower likelihood of seeking dental care among residents of state capitals; this may reflect a combination of factors, such as the greater strain on public services, difficulties in scheduling appointments, and persistent access barriers, even within urbanized settings. This challenges the assumption that geographic proximity alone guarantees access, indicating the need to improve the problem-solving capacity of urban services within SUS. This pattern aligns with data from the study, which pointed to higher use of SUS dental services among older adults residing in municipalities with poorer sanitation conditions and a lower supply of services, characteristics typical of less urbanized areas thereby demonstrating a more equitable demand among the socially most vulnerable groups^17^. Evidence indicates that, in large urban centers, factors such as public network overload, scheduling difficulties, and organizational barriers can reduce the demand for services, even in contexts where supply is abundant^21^. The urban concentration of services does not eliminate inequalities in health-seeking behavior, as organizational and social barriers continue to influence how the population interacts with health systems^1^.

Age, measured in years, showed an inverse association with the demand for dental care, confirming trends documented in the literature regarding the progressive disengagement of older adults from dental services — this a group that frequently prioritizes other health issues over oral care^22^. Factors such as reduced mobility, edentulism, and a perceived low need for treatment are frequently cited as significant barriers within this age group. Data from the SB Brasil 2010 and PNS 2019 surveys corroborate this finding, indicating that the demand for dental services decreases progressively as age advances^12,20^. These findings underscore the urgent need for specific policies regarding oral health care for the older population, such as strengthening home-based care and implementing initiatives tailored to older adults within SUS, particularly given the high prevalence of oral health problems and the low demand for services, even in the presence of pain or a need for prostheses^7,17^.

Female sex maintained a positive association with seeking dental services rather than with actual utilization, as previously described in the original manuscript — indicating that women are more likely to seek care. Studies using data from the PNS and SB Brasil 2010 reinforce that men seek dental services less frequently, which may contribute to a higher burden of oral health conditions in this group^20^. This pattern is widely documented in the literature and reflects cultural norms that foster greater health-seeking behavior among women compared to men, a phenomenon associated with sociocultural factors, a heightened perception of need, and increased engagement with health services throughout life^23^. Data from the present study corroborate this evidence, indicating that men tend to seek dental services less frequently — this is possibly due to symbolic barriers linked to cultural norms of masculinity, which discourage the pursuit of health care. This disparity underscores the need for specific, targeted strategies designed to raise awareness and facilitate men's access to oral health services, thereby reducing inequalities in care and promoting more equitable oral health care for the population^15,24^.

The analysis of educational attainment revealed a clear social gradient: the higher the educational level, the greater the likelihood of seeking dental services. In the final model, individuals with higher education demonstrated a more than threefold increased likelihood of seeking dental care. This association highlights the importance of educational capital both in understanding the relevance of oral care and in the capacity to overcome bureaucratic and logistical barriers when seeking health services^9,15^. This pattern had already been identified in the SB Brasil 2010 and PNS 2019 surveys and remains current, indicating the reproduction of social inequalities in health service-seeking behavior^12,20^.

This same pattern was observed regarding family income: individuals with greater purchasing power access services more frequently, even within a universal public system such as SUS. Families with an income exceeding ten minimum wages were nearly four times more likely to seek care, suggesting that the search for dental services remains contingent upon the ability to absorb indirect costs such as transportation expenses and waiting times, or even the cost of seeking complementary private services. This indicates that socioeconomic factors influence both the perception of the importance of care and the capacity to overcome organizational and indirect financial barriers^9,25,26^. This inequality is extensively documented in recent editions of the SB Brasil survey and remains a persistent issue^7,9,19^.

A finding deserving of special attention was the lower rate of dental service utilization among beneficiaries of the BPC. This group comprises individuals facing situations of extreme socioeconomic and functional inequality, for whom reliance on public health systems does not translate into effective use of services. This phenomenon constitutes a troubling paradox: despite their eligibility for social assistance policies, these individuals remain effectively excluded from accessing dental care. The literature indicates that this segment of the population faces additional barriers, including physical limitations, dependence on caregivers, and a lack of alignment between available services and their specific needs^24,27^. This pattern highlights shortcomings in the comprehensiveness of care and in the intersectoral coordination of public policies, indicating an urgent need for strategies that promote effective and comprehensive access for this population.

Finally, although the variable of color or race did not demonstrate statistical significance in the adjusted model, the observed trends indicate a need for deeper investigation into this dimension. The literature suggests that racial inequalities in oral health are frequently mediated by socioeconomic factors, such as income and educational attainment. Thus, the absence of statistical significance does not rule out the existence of racial inequities; rather, it reinforces the need for intersectional analyses that consider the interplay between race, territory, and social conditions^7,25,28,29^. The lower likelihood of seeking dental services among Indigenous people and individuals who did not report their race may reveal structural gaps in the recognition and reception of historically marginalized groups. This finding underscores the importance of developing approaches that are more sensitive to specific ethno-racial needs and of incorporating methodologies that account for racial intersectionalities within healthcare delivery, with the aim of reducing inequalities and fostering more inclusive and equitable care^7,19,27^.

Despite the scope and relevance of this study, several limitations must be acknowledged. Notably, the SB Brasil 2023 survey covers only permanent private households, thereby excluding populations such as people experiencing homelessness, Indigenous people living in villages, quilombolas, riverine communities, and institutionalized individuals—as explicitly stated in the survey's official technical report. Furthermore, although the survey encompasses urban areas and municipalities in the interior, it is not a survey specifically designed for remote rural areas; this may limit the generalizability of the results to certain population groups. Selection biases and self-reported information may affect data accuracy, while methodological changes across different editions of the survey complicate temporal comparisons^6^. The post-COVID-19 pandemic context, regional inequalities, and limitations regarding data availability may further constrain the generalizability of the findings.

The results of this study reaffirm that the utilization of dental services in Brazil remains strongly conditioned by sociodemographic determinants. The observed inequalities reflect the provision of services, as well as the structural conditions regarding the population's living standards, education, and income. Overcoming these inequities requires the formulation and implementation of effective, equity-oriented public policies that consider the social and structural barriers that still limit universal access to oral health care. This approach is fundamental to promoting more inclusive care, reducing disparities, and advancing the consolidation of the right to health for all population groups.
