# Supplementary material for: Inequalities in the demand for dental services in Brazil: associated factors in the 2023 National Oral Health Survey
Source: Rev Bras Epidemiol. 2026 Jul 20;29(Suppl 1):e260027supl1. doi: 10.1590/1980-549720260027.supl.1 (PMC13403695; doi:10.1590/1980-549720260027.supl.1)
Supplement: Translation

# Translation: Desigualdades na procura por serviços odontológicos no Brasil: fatores associados na Pesquisa Nacional de Saúde Bucal 2023

**DOI:** 10.1590/1980-549720260027.supl.1.2

**Authors:** Fernanda Alencar Rodrigues Pereira, Ana Paula Alves Távora de Oliveira, Maraysa Costa Vieira Cardoso, Christina Cesar Praça Brasil, Geraldo Bezerra da Silva, Mirna Albuquerque Frota, Ana Livia Torres de Sousa, Antonio Augusto Ferreira Carioca

**Affiliations:** I Universidade de Fortaleza, Programa de Pós-graduação em Saúde Coletiva – Fortaleza (CE), Brasil.

**Keywords:** Desigualdades de saúde, Assistência odontológica, Inquéritos de saúde bucal, Serviços de saúde bucal

## Abstract

**Objetivo:**

Analisar os fatores sociodemográficos associados à procura por serviços odontológicos no Brasil, com base na Pesquisa Nacional de Saúde Bucal – SB Brasil 2023.

**Métodos:**

Estudo transversal, de base populacional, com dados de 40.489 indivíduos com 18 anos ou mais. A variável dependente foi a procura por serviço odontológico no último ano. As análises consideraram o desenho amostral complexo, com ponderações e correções. Foram realizadas análises descritivas bivariadas (teste do qui-quadrado) e regressão logística múltipla, estimando-se Odds Ratio (OR) com intervalos de confiança de 95%.

**Resultados:**

Cerca de 58,8% dos entrevistados relataram ter procurado atendimento odontológico no último ano. A maior chance de procura esteve associada a sexo feminino (OR=1,54; IC95% 1,21–1,96), maior escolaridade (superior: OR=1,90; IC95% 1,37–2,63) e renda elevada (>10 SM: OR=3,49; IC95% 1,97–6,17). Por outro lado, a idade em anos (OR=0,99; IC95%: 0,98–0,99) e a residência em capitais (OR=0,69; IC95% 0,56–0,99) estiveram associadas a menor chance de procura por serviços.

**Conclusão:**

Persistem desigualdades na procura por serviços odontológicos no Brasil, influenciadas por escolaridade, renda e fatores de vulnerabilidade. Os achados reforçam a necessidade de políticas públicas direcionadas para reduzir barreiras estruturais e ampliar o acesso equitativo à saúde bucal.

## INTRODUÇÃO

A saúde bucal é entendida como a manutenção do estado funcional e estrutural da boca, dentes e estruturas orofaciais. Ela engloba não apenas a realização de atividades essenciais como falar, comer e respirar, mas também se relaciona com dimensões psicossociais incluindo autoconfiança, bem-estar e capacidade de interagir socialmente, implicando diretamente na qualidade de vida dos indivíduos ao longo do curso de suas vidas^1,2^.

Desde 2005, o Brasil tem avançado na organização da atenção em saúde bucal, com a implementação da Política Nacional de Saúde Bucal (PNSB)^3^ – "Brasil Sorridente". Instituída em 2004, essa política visa promover, prevenir e recuperar a saúde bucal da população por meio da reorganização, reorientação e qualificação dos serviços oferecidos, ampliando o acesso ao tratamento odontológico pelo Sistema Único de Saúde (SUS) para pessoas de todas as faixas etárias. Sobretudo, essa política busca combater as desigualdades no atendimento, ampliando o acesso da população a serviços odontológicos de forma integral, com ênfase na Atenção Primária à Saúde (APS)^4,5^.

Destaca-se que a vigilância à saúde é uma das diretrizes da PNSB^3^, sendo o uso de informações epidemiológicas para orientar o planejamento das ações em saúde bucal um dos pilares dessa política. Nesse contexto, insere-se a Pesquisa Nacional de Saúde Bucal – SB Brasil 2023^3^, cuja análise dos dados permite compreender e identificar não somente o perfil de doenças bucais mais prevalentes, mas também os aspectos sociodemográficos dos usuários e não usuários dos serviços, identificando os fatores associados à procura por atendimento odontológico^4,6^. À medida que proporcionou melhorias no acesso à saúde bucal, a PNSB distancia-se do modelo assistencial de práticas predominantemente curativas e mutiladoras.

A literatura científica brasileira^7–11^ e os inquéritos nacionais, a exemplo da PNSB e da Pesquisa Nacional de Saúde (PNS), registram de forma ampla e consistente as desigualdades no acesso aos serviços odontológicos no país, evidenciando um padrão persistente de maior utilização entre indivíduos com renda e escolaridade mais elevadas, mulheres e usuários do setor privado. Essa realidade conflita com os menores níveis de acesso a esses serviços entre pessoas idosas de baixa renda e em situação de maior vulnerabilidade social, mesmo diante da existência de um sistema de saúde universal^3,12^.

A PNS 2019^12^, em particular, evidenciou relevantes iniquidades socioeconômicas e regionais no uso de serviços odontológicos, reforçando o papel dos determinantes sociais na organização do cuidado em saúde bucal no país. Destaca-se, entretanto, que muitas das informações disponíveis respaldam-se em dados anteriores à pandemia de COVID-19 ou em inquéritos inespecíficos de saúde bucal, limitando a compreensão das desigualdades e da procura por atendimento odontológico em um cenário de profundas mudanças sociais e organizacionais dos serviços.

Observa-se que as desigualdades relacionadas a questões socioeconômicas, sexo, idade e barreiras estruturais regionais têm influência sobre a procura por serviços de odontologia no país^3,9^. Contudo, ainda permanecem pouco explorados o impacto de marcadores diretos de vulnerabilidade social, como o recebimento de benefícios assistenciais, bem como padrões territoriais menos evidentes, como as diferenças entre capitais e municípios do interior.

Nesse sentido, ao utilizar dados da SB Brasil 2023^3^ e analisar a procura por serviços odontológicos nos 12 meses anteriores à entrevista, o presente estudo atualiza o diagnóstico das desigualdades em saúde bucal no Brasil e amplia a análise dos fatores sociodemográficos associados à demanda por atendimento. Ao incorporar marcadores contemporâneos de vulnerabilidade social, como o recebimento do Benefício de Prestação Continuada (BPC)^13^, o estudo avança para além das análises clássicas baseadas apenas em renda e escolaridade e dialoga com evidências provenientes de análises das edições anteriores da SB Brasil e da PNS 2019. Além disso, os resultados indicam menor procura por serviços nas capitais, achado que tensiona interpretações tradicionais sobre oferta e acesso e sugere a persistência de barreiras estruturais mesmo em contextos em que há maior concentração de serviços. Dessa forma, o estudo contribui para o refinamento do debate sobre equidade e oferece subsídios empíricos para o aprimoramento de políticas públicas no âmbito do SUS.

Contudo, ainda há lacuna de estudos recentes que atualizem esses resultados a partir do novo inquérito nacional de saúde bucal, sobretudo com maior abrangência analítica e ajuste de variáveis sociais emergentes, como programas de transferência de renda, interação de determinantes sociais e econômicos e disparidades entre capitais e municípios do interior^14–17^. Diante desse cenário, o presente estudo teve como objetivo analisar os fatores sociodemográficos associados à procura por serviços odontológicos no Brasil, com base nos dados da SB Brasil 2023.

## MÉTODOS

### Desenho do estudo

Trata-se de um estudo epidemiológico transversal, de base populacional e abordagem quantitativa, desenvolvido a partir da análise dos microdados da Pesquisa Nacional de Saúde Bucal – SB Brasil 2023.

### Contexto (*setting*)

A SB Brasil 2023 foi conduzida por meio de parceria entre o Ministério da Saúde e o Departamento de Odontologia Social e Preventiva da Universidade Federal de Minas Gerais (UFMG), com execução técnico-científica entre 2017 e 2024. Consistiu em estudo de base domiciliar, cuja coleta de dados ocorreu durante o período de março de 2022 a março de 2024. As equipes de campo foram compostas por cirurgiões-dentistas (exames epidemiológicos), técnicos ou auxiliares em saúde bucal (registro dos dados) e agentes comunitários de saúde (arrolamento das famílias).

O inquérito seguiu recomendações da Organização Mundial da Saúde (OMS), com estratificação em idades-índice (5, 12, 15–19, 35–44 e 65–74 anos). Foram considerados 53 domínios geográficos (capitais, estados e Distrito Federal), a partir dos quais setores censitários e domicílios foram selecionados aleatoriamente. A amostra final incluiu aproximadamente 50,8 mil participantes, submetidos a entrevistas e exames bucais.

As entrevistas foram conduzidas presencialmente nos domicílios selecionados por equipes treinadas e padronizadas a partir de um questionário estruturado. Este contemplava perguntas relativas ao perfil demográfico e socioeconômico das famílias, como posse de bens duráveis no domicílio, renda familiar, acesso à internet e recebimento de benefícios de programas sociais, bem como características individuais dos participantes, incluindo idade, sexo, cor ou raça e escolaridade. O instrumento também investigava o acesso e a utilização de serviços de saúde bucal, a ocorrência de morbidade bucal autorreferida (dor dentária e dor facial), além da autopercepção da saúde bucal e seu impacto na vida diária.

Antes do início do trabalho de campo, todos os profissionais participaram de capacitações conduzidas pela equipe de coordenação da pesquisa. Nessas oficinas, foram abordados os protocolos de identificação dos participantes, a padronização dos códigos e dos critérios dos exames bucais e o uso do sistema eletrônico de coleta. Além disso, foram realizados monitoramentos periódicos para assegurar a qualidade da coleta e a consistência dos dados obtidos.

Ressalta-se que maiores detalhes metodológicos estão disponíveis no material eletrônico já publicado "SB Brasil 2023: Pesquisa Nacional de Saúde Bucal: relatório final"^6^.

### Participantes

Para o presente estudo, foram considerados apenas indivíduos com idade igual ou superior a 18 anos, totalizando 40.489 participantes com dados válidos para a variável dependente "procura por serviço odontológico no último ano". A análise foi conduzida preservando o delineamento amostral complexo da pesquisa original, com incorporação de pesos amostrais, estratos e conglomerados.

### Variáveis

A variável dependente foi a procura por serviço odontológico, que consistiu na pergunta "No último ano, o(a) sr.(a) (você) procurou algum consultório odontológico, serviço de saúde bucal ou dentista/equipe de saúde bucal para ser atendido?", com as seguintes opções de resposta: "Não procurei"; "Procurei e não fui atendido"; "Procurei e fui agendado para outro dia/outro local"; "Procurei e fui atendido"; "Não sei/não respondeu)". Para fins analíticos, a variável foi operacionalizada de forma dicotômica, considerando-se como "sim" as respostas "Procurei e não fui atendido", "Procurei e fui agendado para outro dia/outro local" e "Procurei e fui atendido", e como "não" a resposta "Não procurei". As respostas "Não sei/não respondeu" foram tratadas como dados faltantes.

As independentes incluíram: região geográfica (Norte, Nordeste, Sudeste, Sul, Centro-Oeste); local de residência (capital da Unidade da Federação – UF)/interior); sexo (masculino, feminino); idade (em anos); cor/raça autorreferida (branca, preta, parda, amarela, indígena); escolaridade (não estudou/fundamental incompleto, fundamental completo, ensino médio e ensino superior) e renda familiar em Salários-mínimos (SM) (Até 1 SM, > 1 a 3 SM, > 3 a 10 SM e > 10 SM). Também foram incluídas variáveis referentes à participação em programas sociais governamentais, consideradas separadamente: recebimento do Programa Bolsa Família (não, sim); recebimento do Benefício de Prestação Continuada – BPC/LOAS (não, sim) e participação em outros programas de transferência de renda (não, sim). As respostas "Não sei/não respondeu" foram tratadas como dados faltantes.

### Métodos estatísticos

As análises foram realizadas no Stata^®^ 17.0, utilizando pesos amostrais e correções para o desenho complexo. Realizou-se análise descritiva para caracterização da amostra e bivariada (teste qui-quadrado de Pearson). Em seguida, aplicou-se regressão logística múltipla com entrada hierarquizada das variáveis, segundo modelo conceitual de Victora et al.^18^, estimando Odds Ratio (OR) brutas e ajustadas com intervalos de confiança de 95%. Foi adotado um modelo conceitual hierárquico para análise dos fatores associados à procura por atendimento odontológico. No nível mais distal (modelo 1) foram incluídas características demográficas individuais (idade e sexo), enquanto no nível intermediário (modelo 2) foram consideradas variáveis socioeconômicas (escolaridade, BPC e renda familiar) e no nível mais proximal (modelo 3) foram incluídas variáveis contextuais relacionadas ao local de residência (grandes regiões do país e residência em capital). As variáveis foram inseridas sequencialmente no modelo, permanecendo nos níveis subsequentes aquelas com significância estatística conforme o modelo hierárquico.

### Aspectos éticos

O estudo seguiu os princípios éticos de pesquisas com seres humanos, tendo sido aprovado pela Comissão Nacional de Ética em Pesquisa (Conep) em 3 de julho de 2021 (CAAE 34497120.6.3001.0008; parecer n° 4.823.054).

#### Declaração de Disponibilidade de Dados:

Todo o conjunto de dados que dá suporte aos resultados deste estudo está disponível mediante solicitação ao Ministério da Saúde. Para ter acesso aos bancos de dados do SB Brasil, é necessário concordar com o termo de compromisso e preencher os dados solicitados pelo Ministério da Saúde.

## RESULTADOS

Dos participantes analisados, 58,8% relataram ter procurado serviços odontológicos nos últimos 12 meses. A Tabela 1 apresenta a distribuição dessa procura segundo diferentes variáveis sociodemográficas, evidenciando padrões de desigualdade no cuidado odontológico no Brasil. A procura foi associada com cidade de residência, idade, sexo, escolaridade, renda familiar e recebimento do Benefício Assistencial de Prestação Continuada.

**Tabela 1:** Distribuição da procura por serviços odontológicos segundo características sociodemográficas. Pesquisa Nacional de Saúde Bucal (2023). Brasil, 2026.

Variáveis		Não procurou (41,2%)	Procurou (58,8%)	p-valor	Total
Grandes regiões					
	Norte	8,1 (6,6–9,9)	7,6 (5,8–10,0)	0,091	7,8 (6,4–9,5)
	Nordeste	27,0 (23,5–30,8)	25,9 (22,1–30,2)		26,4 (23,1–29,9)
	Sudeste	42,1 (36,6–47,8)	42,8 (36,3–49,7)		42,5 (37,0–48,3)
	Sul	16,5 (13,7–19,9)	14,1 (11,5–17,3)		15,1 (12,7–17,9)
	Centro-Oeste	6,3 (4,9–7,9)	9,4 (6,5–13,5)		8,1 (6,0–11,0)
Cidade de residência, capital		26,0 (22,8–29,4)	22,1 (19,1–25,4)	0,014	23,7 (20,9–26,7)
Idade, anos		48,1 (47,1–49,2)	43,5 (42,6–44,3)	<0,001	
Sexo					
	Masculino	41,9 (38,7–45,1)	33,2 (30,9–35,7)	<0,001	36,8 (34,9–38,7)
	Feminino	58,1 (54,9–61,3)	66,8 (64,3–69,1)		63,2 (61,3–65,1)
Cor ou raça					
	Branca	42,2 (38,3–46,2)	44,0 (40,1–47,9)	0,446	43,2 (39,8–46,7)
	Preta	14,5 (12,2–17,1)	12,3 (10,6–14,2)		13,2 (11,6–15,0)
	Amarela	1,1 (0,7–1,6)	1,2 (0,8–2,0)		1,2 (0,8–1,7)
	Parda	40,4 (36,6–44,4)	41,0 (37,4–44,7)		40,8 (37,5–44,1)
	Indígena	0,4 (0,2–0,8)	0,3 (0,2–0,6)		0,3 (0,2–0,6)
Benefício assistencial de Prestação Continuada					
	Não	83,1 (79,4–86,3)	88,2 (84,7–91,0)	0,013	86,1 (83,1–88,7)
	Sim	9,4 (7,3–12,0)	6,0 (4,7–7,6)		7,4 (6,1–8,9)
Benefício assistencial de Programa Bolsa Família					
	Não	75,5 (72,1–78,7)	76,7 (72,6–80,4)	0,667	76,2 (73,0–79,1)
	Sim	17,7 (15,4–20,3)	17,6 (14,9–20,6)		17,6 (15,6–19,9)
Benefício assistencial de outros programas sociais do governo					
	Não	79,8 (76,2–83,0)	81,2 (77,7–84,3)	0,648	80,6 (77,7–83,2)
	Sim	13,2 (11,1–15,7)	12,9 (10,7–15,4)		13,0 (11,2–15,0)
Maior escolaridade					
	Não estudou/fundamental incompleto	34,5 (31,4–37,8)	20,1 (17,8–22,4)	<0,001	26,0 (23,7–28,4)
	Fundamental completo	10,1 (8,4–12,0)	8,3 (6,7–10,2)		9,0 (7,9–10,3)
	Ensino médio	42,0 (38,6–45,4)	47,8 (44,1–51,5)		45,4 (42,6–48,3)
	Ensino superior	13,4 (10,5–17,0)	23,9 (21,0–26,9)		19,6 (17,0–22,5)
Renda familiar					
	Até 1 SM	27,1 (23,1–31,6)	24,3 (20,3–28,9)	0,002	25,6 (21,9–29,5)
	> 1 a 3 SM	52,3 (48,2–56,4)	46,7 (41,7–51,8)		49,0 (45,3–52,7)
	> 3 a 10 SM	20,0 (16,0–24,7)	26,9 (23,1–31,2)		24,1 (21,0–27,6)
	> 10 SM	0,6 (0,4–0,9)	2,0 (1,3–3,2)		1,4 (0,9–2,1)

IC95%: Intervalo de confiança de 95%. Teste de qui-quadrado de Pearson para variáveis categóricas. Dados ponderados e ajustados para o desenho amostral complexo.

Fonte: Brasil, SB Brasil 2023.

Na Tabela 2, os moradores da região Centro-Oeste apresentaram maior chance de procura (OR=1,59; IC95% 1,04–2,44) em comparação com os da região Norte, porém morar em capitais reduziu essa chance (OR=0,80; IC95% 0,68–0,96). A idade em anos diminuiu as chances do desfecho (OR=0,98; IC95% 0,98–0,99), e o sexo feminino aumentou a chance de procura (OR=1,44; IC95% 1,21–1,73) em relação aos homens. A variável cor ou raça não foi associada com o desfecho.

**Tabela 2:** Análise bivariada dos fatores associados à procura por atendimento odontológico. Pesquisa Nacional de Saúde Bucal 2023. Brasil, 2026.

Variáveis independentes		Variável dependente: procura por atendimento odontológico		
			OR	IC95%	
Grandes regiões				
	Norte	Referência		
	Nordeste	1,01	0,72	1,44
	Sudeste	1.07	0,74	1,57
	Sul	0,90	0,74	1,57
	Centro-Oeste	1,59	1,04	2,44
Cidade de residência, capital		0,80	0,68	0,96
Idade (anos)		0,98	0,98	0,99
Sexo				
	Masculino	Referência		
	Feminino	1,44	1,21	1,73
Cor ou raça				
	Branca	Referência		
	Parda	0,82	0,65	1,02
	Amarela	1,12	0,61	2,07
	Parda	0,97	0,82	1,16
	Indígena	0,74	0,33	1,70
Benefício assistencial de Prestação Continuada				
	Não	Referência		
	Sim	0,60	0,42	0,86
Benefício assistencial de Programa Bolsa Família				
	Não	Referência		
	Sim	0,97	0,78	1,23
Benefício Assistencial de outros programas sociais do governo				
	Não	Referência		
	Sim	0,95	0,74	1,22
Maior escolaridade				
	Não estudou/fundamental incompleto	Referência		
	Fundamental completo	1,42	1,06	1,90
	Ensino médio	1,96	1,61	2,39
	Ensino superior	3,08	2,37	3,99
Renda familiar				
	Até 1 SM	Referência		
	> 1 a 3 SM	1,00	0,78	1,27
	> 3 a 10 SM	1,50	1,12	2,01
	> 10 SM	3,96	2,34	6,71

Odds Ratio (OR) brutas com respectivos intervalos de confiança de 95% (IC95%), obtidas por regressão logística bivariada. Análises ponderadas e ajustadas para o desenho amostral complexo da Pesquisa Nacional de Saúde Bucal – SB Brasil 2023.

Fonte: Brasil, SB Brasil 2023.

Destaca-se que o recebimento do BPC reduziu significativamente a chance de busca por atendimento (OR=0,60; IC95% 0,42–0,86), ao passo que o Bolsa Família e outros benefícios não apresentaram associação estatística. Ademais, a escolaridade revelou um gradiente importante: ensino médio (OR=1,96; IC95% 1,61–2,39) e superior completo (OR=3,08; IC95% 2,37–3,99) aumentaram significativamente a chance de procura em relação a quem nunca estudou.

A renda familiar manteve padrão semelhante à escolaridade: indivíduos com mais de 10 SM apresentaram quase quatro vezes mais chance de procurar atendimento (OR=3,96; IC95% 2,34–6,71) em comparação à renda familiar de até 1 SM.

Na Tabela 3, no modelo final, os fatores independentemente associados à maior probabilidade de procurar serviços odontológicos foram: sexo feminino (OR=1,54; IC95% 1,21–1,96); maior escolaridade, especialmente ensino superior (OR=1,90; IC95% 1,37–2,63) e renda familiar superior a 10 salários-mínimos (OR=3,49; IC95% 1,97–6,17). Por outro lado, idade em anos (OR=0,99; IC95% 0,98–0,99) e residir em capitais (OR=0,69; IC95% 0,57–0,84) estiveram associados à menor chance de procura por serviços odontológicos.

**Tabela 3:** Modelo final de regressão logística dos fatores associados à procura por atendimento odontológico. Pesquisa Nacional de Saúde Bucal 2023. Brasil, 2026.

		**Variável dependente: procura por atendimento odontológico**								
		Modelo 1 (sexo e idade)			Modelo 2 (modelo 1 + escolaridade, renda e BPC)			Modelo 3 (modelo 2 + grandes regiões + cidade de residência)		
Variáveis independentes		OR	IC95%		OR	IC95%		OR	IC95%	
Idade (anos)		0,98	0,98	0,99	0,97	0,98	0,99	0,99	0,98	0,99
Sexo										
	Masculino	Referência			Referência			Referência		
	Feminino	1,45	1,20	1,75	1,54	1,20	1,96	1,54	1,21	1,96
Maior escolaridade										
	Não estudou/fundamental incompleto				Referência			Referência		
	Fundamental completo				1,18	0,82	1,71	1,20	0,84	1,73
	Ensino médio				1,38	1,08	1,76	1,42	1,11	1,82
	Ensino superior				1,84	1,34	2,55	1,90	1,37	2,63
Renda familiar										
	Até 1 SM				Referência			Referência		
	> 1 a 3 SM				0,97	0,77	1,23	1,02	0,82	1,26
	> 3 a 10 SM				1,34	0,98	1,84	1,47	1,08	1,98
	> 10 SM				3,03	1,70	5,41	3,49	1,97	6,17
Benefício assistencial de Prestação Continuada										
	Não						Referência			Referência		
	Sim				0,71	0,45	1,14	0,71	0,44	1,14
Grandes regiões										
	Norte							Referência		
	Nordeste							1,07	0,70	1,65
	Sudeste							0,96	0,61	1,50
	Sul							0,76	0,47	1,22
	Centro-Oeste							1,40	0,84	2,34
Cidade de residência, capital								0,69	0,57	0,84

Odds Ratio (OR) ajustadas e intervalos de confiança de 95% (IC95%), com base em modelo hierarquizado. As variáveis foram ajustadas entre si de acordo com blocos de determinação social. Análises ponderadas para o delineamento amostral complexo da SB Brasil 2023.

Fonte: Brasil, SB Brasil 2023.

## DISCUSSÃO

Os achados deste estudo reforçam a persistência de desigualdades estruturais na procura por serviços odontológicos no Brasil, mesmo diante de avanços institucionais promovidos pela Política Nacional de Saúde Bucal. Esses resultados se alinham ao que foi relatado por diversos autores, que destacam o papel dos determinantes sociais da saúde no padrão de procura por serviços odontológicos, especialmente em países com sistemas de saúde universais e desiguais como o Brasil^15,17,19^.

A proporção de 58,8% dos entrevistados que relataram ter procurado atendimento nos últimos 12 meses deve ser interpretada como um indicador do comportamento de busca por serviço, condicionado por necessidades percebidas e barreiras estruturais, e não como um parâmetro de cobertura do sistema de saúde, uma vez que não existem metas normativas estabelecidas para esse desfecho no Brasil^6^. Esse percentual deve ser compreendido como um indicador descritivo do comportamento de busca, condicionado por necessidades percebidas, barreiras estruturais, características individuais e contextuais. Resultados semelhantes foram observados na Pesquisa Nacional de Saúde (PNS) 2019 e em análises do SB Brasil 2010^12,20^, que demonstraram que parcela expressiva da população não procura atendimento odontológico regularmente, mesmo em um sistema universal de saúde^7,9^. A distribuição desigual entre regiões, apesar de não ter alcançado significância estatística, revela concentrações importantes da demanda no Sudeste e no Nordeste, refletindo tanto a densidade populacional quanto a estrutura histórica de alocação dos serviços no país. Estudos recentes reforçam que a localização dos serviços e a capacidade instalada variam fortemente entre regiões, o que impacta diretamente o acesso e o uso da atenção em saúde bucal^7,15^.

Um achado relevante e contraintuitivo foi a menor chance de procura por atendimento odontológico entre residentes das capitais, que pode refletir uma combinação de fatores, como maior sobrecarga dos serviços públicos e dificuldade de agendamento e barreiras de acesso mesmo em contextos urbanizados. Isso desafia o pressuposto de que a proximidade geográfica por si só garante acesso, indicando a necessidade de melhorar a resolutividade dos serviços urbanos no SUS. Esse padrão está em consonância com os dados do estudo que apontaram maior uso dos serviços odontológicos do SUS entre idosos residentes em municípios com piores condições de saneamento e menor oferta de serviços, típicos de áreas menos urbanizadas, mostrando uma procura mais equânime entre os mais vulneráveis socialmente^17^. Evidências indicam que, em grandes centros urbanos, fatores como sobrecarga da rede pública, dificuldades de agendamento e barreiras organizacionais podem reduzir a procura por serviços, mesmo em contextos de maior oferta^21^. A concentração urbana de serviços não elimina desigualdades no comportamento de busca, uma vez que barreiras organizacionais e sociais continuam a influenciar a interação da população com os sistemas de saúde^1^.

A idade em anos apresentou associação inversa com a procura por atendimento odontológico, confirmando tendências documentadas na literatura acerca do distanciamento progressivo pelos mais velhos dos serviços odontológicos, que frequentemente priorizam outros problemas de saúde em detrimento do cuidado bucal^22^. Fatores como mobilidade reduzida, edentulismo e percepção de baixa necessidade de tratamento são frequentemente citados como barreiras relevantes nessa faixa etária. Dados do SB Brasil 2010 e da PNS 2019 corroboram com esse achado, apontando que a procura por serviços odontológicos diminui progressivamente com o avanço da idade^12,20^. Esses achados reforçam a urgência de políticas específicas para a atenção à saúde bucal da população idosa, como o fortalecimento da atenção domiciliar e ações direcionadas à terceira idade no SUS, especialmente frente à alta prevalência de problemas bucais e à baixa procura por serviços mesmo na presença de dor ou necessidade de prótese^7,17^.

O sexo feminino manteve associação positiva com a procura por serviços odontológicos, e não com o uso efetivo, como previamente descrito no manuscrito original, com as mulheres apresentando maior probabilidade de buscar atendimento. Estudos com dados da PNS e do SB Brasil 2010 reforçam que homens apresentam menor procura por serviços odontológicos, o que pode contribuir para maior carga de agravos bucais nesse grupo^20^. Esse padrão é amplamente documentado na literatura e reflete normas culturais que promovem uma maior atenção feminina à saúde em comparação aos homens, associado a fatores socioculturais, à maior percepção de necessidade e à maior interação com os serviços ao longo do ciclo de vida^23^. Dados do presente estudo corroboram essas evidências, indicando que os homens tendem a apresentar menor procura pelos serviços odontológicos, possivelmente em decorrência de barreiras simbólicas relacionadas aos padrões culturais de masculinidade, que desencorajam a procura por cuidados de saúde. Essa disparidade ressalta a necessidade de estratégias específicas e direcionadas que possam sensibilizar e facilitar o acesso dos homens aos serviços de saúde bucal, reduzindo, assim, as desigualdades no cuidado e promovendo uma atenção mais equitativa à saúde bucal da população^15,24^.

A análise da escolaridade revelou um claro gradiente social: quanto maior for o nível educacional, maior a probabilidade de procura por serviços odontológicos. Indivíduos com ensino superior apresentaram chance de procurar atendimento odontológico mais de três vezes maior no modelo final. Essa associação evidencia a importância do capital educacional tanto na compreensão da relevância do cuidado bucal quanto na capacidade de superar barreiras burocráticas e logísticas na busca pelo serviço de saúde^9,15^. Esse padrão já havia sido identificado no SB Brasil 2010 e na PNS 2019, e permanece atual, indicando a reprodução de desigualdades sociais no comportamento de procura por serviços^12,20^.

Esse mesmo padrão foi observado com a renda familiar: indivíduos com maior poder aquisitivo acessam os serviços com mais frequência, mesmo em um sistema público universal como o SUS. Famílias com renda superior a dez SM apresentaram quase quatro vezes mais chances de procurar atendimento, sugerindo que a busca por serviços odontológicos ainda está condicionada à capacidade de superar custos indiretos como transporte, tempo de espera ou até a busca por serviços privados complementares, indicando que fatores socioeconômicos influenciam tanto a percepção da importância do cuidado quanto a capacidade de superar barreiras organizacionais e financeiras indiretas^9,25,26^. Essa desigualdade está amplamente documentada nas últimas edições da SB Brasil e permanece atual^7,9,19^.

Um achado que merece atenção especial foi a menor procura por serviços odontológicos entre beneficiários do BPC. Esse grupo representa indivíduos em situação de extrema desigualdade socioeconômica e funcional, cuja dependência dos sistemas públicos de saúde não se traduz em uso efetivo dos serviços. Tal fenômeno configura um paradoxo preocupante: apesar da elegibilidade para políticas assistenciais, essas pessoas permanecem distantes do acesso ao cuidado odontológico. A literatura evidencia que essa parcela da população enfrenta barreiras adicionais, incluindo limitações físicas, dependência de cuidadores e inadequação da oferta de serviços às suas necessidades específicas^24,27^. Esse padrão evidencia falhas na integralidade do cuidado e na articulação intersetorial das políticas públicas, indicando a necessidade urgente de estratégias que promovam o acesso efetivo e integral a essa população.

Por fim, embora a variável cor ou raça não tenha apresentado significância estatística no modelo ajustado, as tendências observadas indicam a necessidade de aprofundamento dessa dimensão. A literatura sugere que desigualdades raciais na saúde bucal são frequentemente mediadas por fatores socioeconômicos, como renda e escolaridade. Assim, a ausência de significância estatística não exclui a existência de iniquidades raciais, mas reforça a necessidade de análises interseccionais que considerem a interação entre raça, território e condições sociais^7,25,28,29^. A menor probabilidade de busca pelos serviços odontológicos entre indígenas e indivíduos que não informaram sua raça pode revelar lacunas estruturais no reconhecimento e acolhimento de grupos historicamente marginalizados. Essa constatação ressalta a importância de desenvolver abordagens mais sensíveis às especificidades étnico-raciais e de incorporar metodologias que considerem as interseccionalidades raciais no cuidado em saúde, visando reduzir as desigualdades e promover um atendimento mais inclusivo e equitativo^7,19,27^.

Apesar da abrangência e da relevância do estudo, algumas limitações devem ser consideradas. Entre elas, destaca-se que a SB Brasil 2023 contempla apenas domicílios particulares permanentes, não incluindo populações tais como pessoas em situação de rua, indígenas aldeados, quilombolas, ribeirinhos e indivíduos institucionalizados, conforme explicitado no relatório técnico oficial da pesquisa. Além disso, embora a pesquisa abranja áreas urbanas e municípios do interior, não se trata de um inquérito específico para áreas rurais remotas, o que pode limitar a generalização dos resultados para determinados grupos populacionais. Vieses de seleção e informações autorreferidas podem afetar a precisão dos dados, enquanto alterações metodológicas entre edições dificultam comparações temporais^6^. O contexto pós-pandemia de COVID-19, as desigualdades regionais e as limitações na disponibilidade de dados podem limitar a generalização dos achados.

Os resultados deste estudo reafirmam que a procura por serviços odontológicos no Brasil permanece fortemente condicionada por determinantes sociodemográficos. As desigualdades observadas refletem a oferta dos serviços, bem como as condições estruturais de vida, educação e renda da população. A superação dessas iniquidades exige a formulação e a implementação de políticas públicas efetivas e orientadas à equidade, que considerem as barreiras sociais e estruturais que ainda limitam o acesso universal à saúde bucal. Esse enfoque é fundamental para promover um atendimento mais inclusivo, reduzir disparidades e avançar na consolidação do direito à saúde para todos os grupos populacionais.
